# Characterization of LhSorTGA2, a novel TGA2-like protein that interacts with LhSorNPR1 in oriental hybrid lily Sorbonne

**DOI:** 10.1186/s40529-017-0201-y

**Published:** 2017-11-10

**Authors:** Le Wang, Zhihong Guo, Yubao Zhang, Yajun Wang, Li Wang, Guo Yang, Wenmei Li, Ruoyu Wang, Zhongkui Xie

**Affiliations:** 1 Gaolan Station of the Agricultural and Ecological Experiment, Northwest Institute of Eco-Environment and Resources, Chinese Academy of Sciences, Lanzhou, 730000 China; 2 University of Chinese Academy of Sciences, Beijing, 100049 China; 3 The Forest Tree Seedling Station of the Alxa League, Alxa League, 750300 China

**Keywords:** Non-expressor of pathogenesis-related genes 1, TGA-like proteins, Protein interaction, Gene expression, Subcellular localization, *Lilium* spp.

## Abstract

**Background:**

Non-expressor of pathogenesis-related genes 1 (NPR1) regulates expression of *pathogenesis*-*related (PR)* genes by interacting with TGA family proteins during systemic acquired resistance (SAR). However, no TGA-like proteins or their interacting partners have been characterized in lily.

**Results:**

In the present study, LhSorTGA2, a novel TGA-like protein, was identified as an interacting partner of LhSorNPR1 (an NPR-like protein) by bimolecular fluorescence complementation (BIFC) and yeast two-hybrid assay (Y2H). Subcellular localization of GFP-tagged proteins targeted LhSorTGA2 to the nucleus, whereas GFP-labeled LhSorNPR1 was observed both in the nucleus and at the cytomembrane. Sequence alignment revealed that LhSorTGA2 was featured with a basic leucine zipper (bZIP) domain and two glutamine rich acid domains (QI and QII). Further phylogenetic analysis showed that TGA family proteins can be grouped into three subclades, within which LhSorTGA2 was clustered into subclade I, together with AtTGA2/5/6. Expression of *LhSorTGA2* was investigated in different tissues by qPCR, and the highest expression level was observed in stem. Besides, when treated with phytohormones (SA, MeJA, ETH and ABA) or fungal pathogen *Botrytis elliptica*, *LhSorTGA2* expression was also induced at different time points post treatments.

**Conclusions:**

Collectively, these results suggested that LhSorTGA2 was an interacting partner of LhSorNPR1, which might function in regulating expression of *PR* genes in lily during SAR.

**Electronic supplementary material:**

The online version of this article (10.1186/s40529-017-0201-y) contains supplementary material, which is available to authorized users.

## Background

Infections at local sites often induce resistance against further invasions of pathogenic organisms in the distal uninfected parts of plants. This primed resistance, which protects plants from further invasion of a broad spectrum of pathogens at the whole-plant level, is referred to as systemic acquired resistance (SAR) (Fu and Dong 2013). The establishment of SAR is accompanied by accumulation of salicylic acid (SA) (Gao et al. 2015), and induction of *pathogenesis*-*related (PR)* genes. *PRs* encode for small proteins, of which some are endowed with direct anti-microbial activities. Since SA level, PR accumulation, and SAR are tightly linked, SAR-conferred resistance could be severely compromised either by interfering the level of SA synthesis or by disrupting signaling pathways control *PR* gene expression (Gaffney et al. 1993). Mutation of the *non*-*expressor of PR genes 1 (NPR1)* gene, a central node in SA-mediated defense signaling, blocks the priming of SAR (Cao et al. 1997). NPR1 lacks a DNA binding domain itself, but features with an ankyrin repeat domain and a BTB/POZ domain, both of which mediate protein–protein interaction (Pieterse and Van Loon 2004). The ankyrin repeat at C-terminus interacts with TGA2 to activate *PR1* transcription, which is critical to plant defense against biotrophic pathogens in the state of SAR (Fan and Dong 2002; Zhang et al. 1999).

TGA proteins are members of the group D basic region/leucine zipper (bZIP) transcription factors (TFs) (Jakoby et al. 2002), which recognize the TGACG motif (also known as *activation sequence 1; as*-*1*) in the promoter region (Gatz 2013). With the identification of as-1 elements in promoters of a number of genes, the crucial roles TGA TFs played in gene expression regulation were unveiled. Mutagenesis of *LS7* element, which contained a TGA-binding site, resulted in complete abolishment of *PR1* expression. On the contrary, mutation of *LS5*, another TGACG motif containing element, augmented *PR1* expression (Kesarwani et al. 2007). The opposite effects exerted by these two TGACG motifs to *PR1* expression implied that transcription of genes containing *as*-*1* elements in the adjacent promoter, might subject to complex regulation by different TGAs in the genome.

Lilies are herbaceous perennials that renowned for their elegant flowers. However, bulb and cut flower production in genus *Lilium*, are both under severe threat of pathogens in different types. For instance, lily bulbs can be infected by some soil-borne fungal pathogens (*Fusarium oxyporum*, *Penicillium albocoremium*, and *Penicillium tulipae*), causing rot of scales, bulb bases, and buds (Lecomte et al. 2016; Wang et al. 2017a). Furthermore, infection of air-borne fungi, such as *Botrytis cinerea* and *Botrytis elliptica*, could lead to fire blight disease, which is destructive to cut flower production (Huang et al. 2012; Wang et al. 2017d). In addition, multiplication of viruses (lily symptomless virus, cucumber mosaic virus, and lily mottle virus) also affects plant growth, resulting in leaf mottle and contortion, as well as stunted growth of lily plants (Zhang et al. 2016). To counteract pathogenic organisms, a comprehensive understanding of defense related genes that govern plant immunity in lily would be of great necessity.

In the present study, LhSorTGA2, a TGA2-like protein that interacted with LhSorNPR1 (Wang et al. 2017b) was characterized. Subcellular localization revealed that presence of GFP-tagged LhSorNPR1 was detected both in the nucleus and at the cytomembrane, but the GFP-tagged LhSorTGA2 was predominantly detected in the nucleus. *LhSorTGA2* expression in response to various phytohormone treatments and fungal pathogen *B. elliptica* infection was also explored.

## Methods

### Plant material and treatments

Bulbs (10–12 cm in diameter) of oriental hybrid cultivar ‘Sorbonne’ were planted in plastic pots containing peat moss as a growing substrate. All pots were then transferred to the growth chamber of Gaolan Station of Agricultural and Ecological Experiment, to keep plants under controlled conditions (16 h light/8 h dark; 22 °C). *Nicotiana benthamiana* plants were raised under the same conditions for observation of protein localization in planta.

### Full-length cloning of *LhSorTGA2*

Total RNA was extracted from the leaves of 1-month-old seedlings using the RNAprep Pure Kit for plants (TIANGEN Corporation, Beijing, China). Partial coding sequence of *LhSorTGA2*, which was annotated in our previous RNA-seq data, was used to design gene specific primers (5RACE-1, 5RACE-2, 3RACE-1, 3RACE-2; Table 1) for rapid amplification of cDNA ends (RACE). 5′ RACE and 3′ RACE were conducted according to the instructions of the SMARTer^®^ RACE 5′/3′ Kit (Takara, China). The cDNA full-length sequence of *LhSorTGA2* was obtained by assembling the 5′-RACE sequence, the partial coding sequence, and the 3′-RACE sequence.

**Table 1:** List of primers used in the experiment

Experiment	Primer name	Primer sequence (5′–3′)
LhSorTGA2 5′ RACE	LhSorTGA2-5RACE-1	CAGGTCAGCAGCATCCTCAATTCGG
	LhSorTGA2-5RACE-2	ATAGCCCCGAAGTCAGAAACCCGGG
LhSorTGA2 3′ RACE	LhSorTGA2-3RACE-1	GAACCTCCAGCAGTCCTCCCAGCAG
	LhSorTGA2-3RACE-2	GGGTCAGATGGCTATGGCGATGGGA
BIFC	LhSorTGA2-BIFC-F	CGTCTAGAATGCACTCCTCCCGGGTTTCT
	LhSorTGA2-BIFC-R	CGGGTACCTTCCCGTGGACGGGCTAGCCA
	LhSorNPR1-BIFC-F	CGGGGCCCATGGCCGACGCCGCCGAGTG
	LhSorNPR1-BIFC-R	CGGGATCCTCATTCTTCCATATCTACCAGACA
Y2H	LhSorTGA2-GADT7-F	CGGAATTCATGCACTCCTCCCGGGTTTCT
	LhSorTGA2-GADT7-R	CGGGATCCCTCATTCCCGTGGACGGGCTAG
	LhSorNPR1-GBKT7-F	CGCCATGGAGATGGCCGACGCCGCCGA
	LhSorNPR1-GBKT7-R	CGGGATCCCTCATTCTTCCATATCTACCAGACAG
Subcellular localization	LhSorNPR1-sub-F	ATATCCATGGTAATGGCCGACGCCGCCGA
	LhSorNPR1-sub-R	CGACTAGTTTCTTCCATATCTACCAGACAGACA
	LhSorTGA2-sub-F	CGACTAGTATGCACTCCTCCCGGGTTTCTGA
	LhSorTGA2-sub-R	CGACTAGTTTCCCGTGGACGGGCTAGCCA
LhSorTGA2 qPCR	LhSor TGA2 qPCR-F	TCAGATGGCTATGGCGATGG
	LhSor TGA2 qPCR-R	CGGATTGGCGAGTAGTGAGT

Restriction sites for vector construction are underlined

### In silico analysis of LhSorTGA2

The open reading frame (ORF) of the assembled *LhSorTGA2* full-length cDNA was predicted by ORF finder. The theoretical isoelectric point and molecular weight were calculated by using the Compute pI/Mw tool of ExPASy. Sequences of TGA proteins were aligned with the Clustal W program. Construction of phylogenetic tree was carried out by Mega 5 software using the neighbor-joining method (Tamura et al. 2011).

### Subcellular localization and protein–protein interaction validated by BIFC

The ORF of *LhSorTGA2* without stop codon was amplified using primers LhSorTGA2-sub-F and LhSorTGA2-sub-R, and inserted into *Spe*I digested pCAMBIA1302 vector to express LhSorTGA2 in frame with GFP at the N-terminus. For the vector construction of LhSorNPR1, the ORF of *LhSorNPR1* was amplified with primers LhSorNPR1-sub-F and LhSorNPR1-sub-R, and cloned into *Nco*I and *Spe*I digested pCAMBIA1302 vector after sequencing. The recombinant plasmids pCAMBIA1302-LhSorTGA2 and pCAMBIA1302-LhSorNPR1 were each introduced into *Agrobacterium tumefaciens* (strain GV3101) competent cells via the freeze-thaw method. GV3101 cells harbouring pCAMBIA1302-LhSorTGA2 or pCAMBIA1302-LhSorNPR1 construct were infiltrated into leaf epidermal cells of 4-week-old *N. benthamiana* plants using a 1-ml needleless syringe. The pCAMBIA1302 empty vector was used as the control for protein localization.

To validate the protein–protein interaction between LhSorTGA2 and LhSorNPR1, the bimolecular fluorescence complementation (BiFC) assay was performed using vectors described previously. *LhSorNPR1* was constructed into the pSPYNE(R)173 vector to express LhSorNPR1 in fusion with the N-terminus of YFP (LhSorNPR1-YFP^N^), and *LhSorTGA2* was inserted into the pSPYCE(M) vector to express LhSorTGA2 in fusion with the C-terminus of YFP (LhSorTGA2-YFP^C^). The resulting recombinant plasmids, pSPYNE-LhSorNPR1 and pSPYCE-LhSorTGA2, were transformed into GV3101 and co-infiltrated into *N. benthamiana* leaf epidermal cells using a protocol described previously (Kerppola 2013). Fluorescence of YFP or GFP was observed 72 h post infiltration using a confocal laser scanning microscope (Leica TCS SP8, Leica Microsystems, Germany).

### Yeast two-hybrid assay

To further confirm the protein interaction between LhSorTGA2 and LhSorNPR1, the ORF of *LhSorNPR1* was amplified (LhSorNPR1-GBKT7-F, LhSorNPR1-GBKT7-R; Table 1) and inserted into pGBKT7 vector to construct the pGBKT7-LhSorNPR1 bait plasmid. The prey plasmid, pGADT7-LhSorTGA2, was generated by cloning the *LhSorTGA2* ORF into the pGADT7 vector after amplification with primers (LhSorTGA2-GADT7-F, LhSorTGA2-GADT7-R; Table 1). Constructed vectors were co-transformed into AH109 competent yeast cells in pairs, and transformants were plated on DDO and QDO mediums to test protein interactions. Transformants were also streaked on QDO medium supplemented with 40 µg/ml 5-bromo-4-chloro-3-indoxyl-α-d-galactopyranoside (X-α-Gal) to further confirm interaction of different co-transformants.

### Gene expression analysis by qPCR

For tissue-specific expression of *LhSorTGA2*, root, stem, leaf, petal, and scale were sampled from 2-month-old fully bloomed plants and subjected to total RNA isolation as described above. For *LhSorTGA2* expression in respond to hormone or pathogen treatment, lily seedlings of 1-month-old were subjected to 20 mM methyl jasmonate (MeJA), 10 mM sodium salicylate (SA), 5 mM ethephon (ETH), 2 µM abscisic acid (ABA) or *B. elliptica* (8 × 10^5^ conidia/ml) inoculation treatments. Preparation of phytohormone solutions, the *B. elliptica* inoculum, and the corresponding mock treatments were performed according our previous report (Wang et al. 2017a). Leaves were sampled at 0, 2, 4, 8, 12, and 24 h post treatments, and froze immediately in liquid nitrogen and kept in a refrigerator at − 80 °C) until use.

After RNA extraction, 500 ng of total RNA was reversely transcribed to cDNA with the HiScript II Q RT SuperMix for qPCR kit (Vazyme Biotech, Nanjing, China). *LhSorTGA2* specific primers (LhSorTGA2 qPCR-F, LhSorTGA2 qPCR-R; Table 1) were used for qPCR amplification, and the housekeeping gene *polyubiquitin 4* was used as an internal reference (Wang et al. 2017c). All PCR reactions were conducted in a MX3000P qPCR thermocycler system (Stratagene Corp., USA). The following protocol was used for amplification: pre-denaturation for 10 min at 95 °C, followed by 40 cycles of 94 °C for 15 s, 55 °C for 15 s, and 72 °C for 30 s. The relative expression software tool (REST) was used for gene expression data analysis, and we applied P(H1) testing for statistical analysis. The Origin Pro 8.1 software was used for data graphing.

## Results

### Molecular cloning and sequence analysis of *LhSorTGA2*

The partial coding sequence of *LhSorTGA2* was retrieved from sequences annotated in our previous RNA-seq data. Gene specific primers were designed for 5′ RACE and 3′ RACE PCR to obtain the *LhSorTGA2* full-length sequence. DNA fragments amplified during RACE cloning were shown in Additional file 1: Figure S1. The *LhSorTGA2* cloned was 2010 bp in length, comprising a 5′ untranslated region (UTR) of 337 bp, a predicted open reading frame (ORF) of 1278 bp, and a 3′ UTR of 395 bp. The ORF encoded a protein of 425 amino acids with a theoretical molecular mass of 47.34 kDa and a predicted isoelectric point (pI) of 6.74. The *LhSorTGA2* full-length cDNA sequence has been submitted to GenBank and deposited under the Accession Number MF685037.

**Fig. 1:**
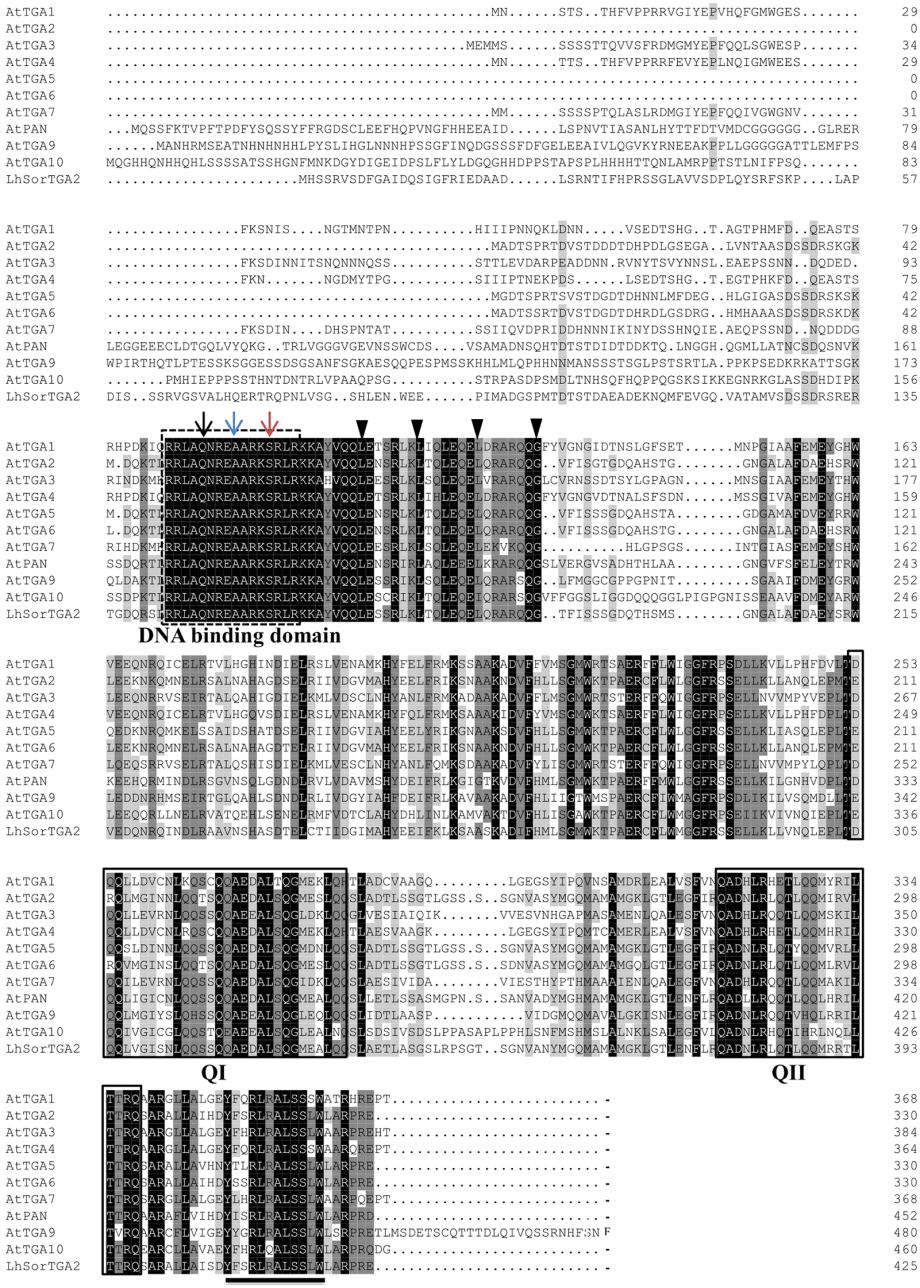
Sequence alignment of LhSorTGA2 with proteins of the TGA family in *Arabidopsis*. Sequences were aligned using Clustal W. Identical residues are shaded in black, highly similar residues are shaded in dark gray, and similar residues are shaded in gray. The DNA binding domain (DBD) is indicated by a dashed line box. The conserved glutamine residues (black arrow), alanine residues (blue arrow) and serine residues (red arrow), are indicated by arrows. Three leucine residues and one glycine residue for the four heptad repeats in leucine zipper are highlighted by triangles. Two glutamine rich acid domains (QI and QII) that mediate transcription activation are highlighted by solid line boxes. The signature sequence for group D bZIP transcription factors, Yx_2_RL[RQ]ALSS[LS]W, is underlined. The accession numbers of all the TGA proteins used for sequence alignment are listed in Additional file 2: Table S1

**Fig. 2:**
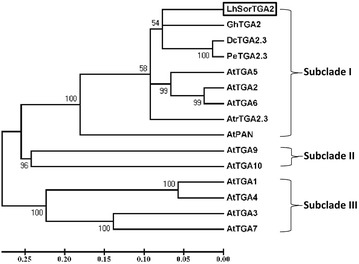
Phylogenetic relationship of LhSorTGA2 with TGA family proteins from other plants. The phylogram was constructed using the neighbor-joining method in Mega 5, and tested with 1000 bootstrap iterations. The Accession Numbers of all the TGA proteins used for phylogram construction are listed in Additional file 2: Table S1

Alignment of LhSorTGA2 protein sequence to TGAs in *Arabidopsis thaliana* revealed that LhSorTGA2 was highly similar to members of the TGA family protein (Fig. 1). LhSorTGA2 contained the feature structures of bZIP transcription factors: a DNA binding domain (DBD) and a leucine zipper that enables protein dimerization. Within the DBD, glutamine, alanine, and serine residues, which were highly conserved for all TGA homologues were identified. Phosphorylation of the serine residue within the DBD led to DNA binding impediment, suggesting that phosphorylation might involve in the fine tune of transcription control of LhSorTGA2-regulated downstream genes (Kirchler et al. 2010). Leucine zippers of TGA proteins were formed by four heptads, within which three conserved leucine residues and one glycine residue were identified (Fig. 1). Two glutamine rich acid domains (QI and QII), which functioned in transcription activation, were also found in LhSorTGA2. The signature sequence for group D bZIP transcription factors, Yx_2_RL[RQ]ALSS[LS]W, was identified at the C-terminus of all TGA homologues. A phylogenetic tree was built to analyze the evolutionary relationship of LhSorTGA2 with other TGA family proteins in plants. As shown in Fig. 2, TGA family proteins were subdivided into three clades, and LhSorTGA2 was most closely related to GhTGA2 in subclade I.

### Subcellular localization of LhSorTGA2 and LhSorNPR1, and protein–protein interaction validated by BIFC

**Fig. 3:**
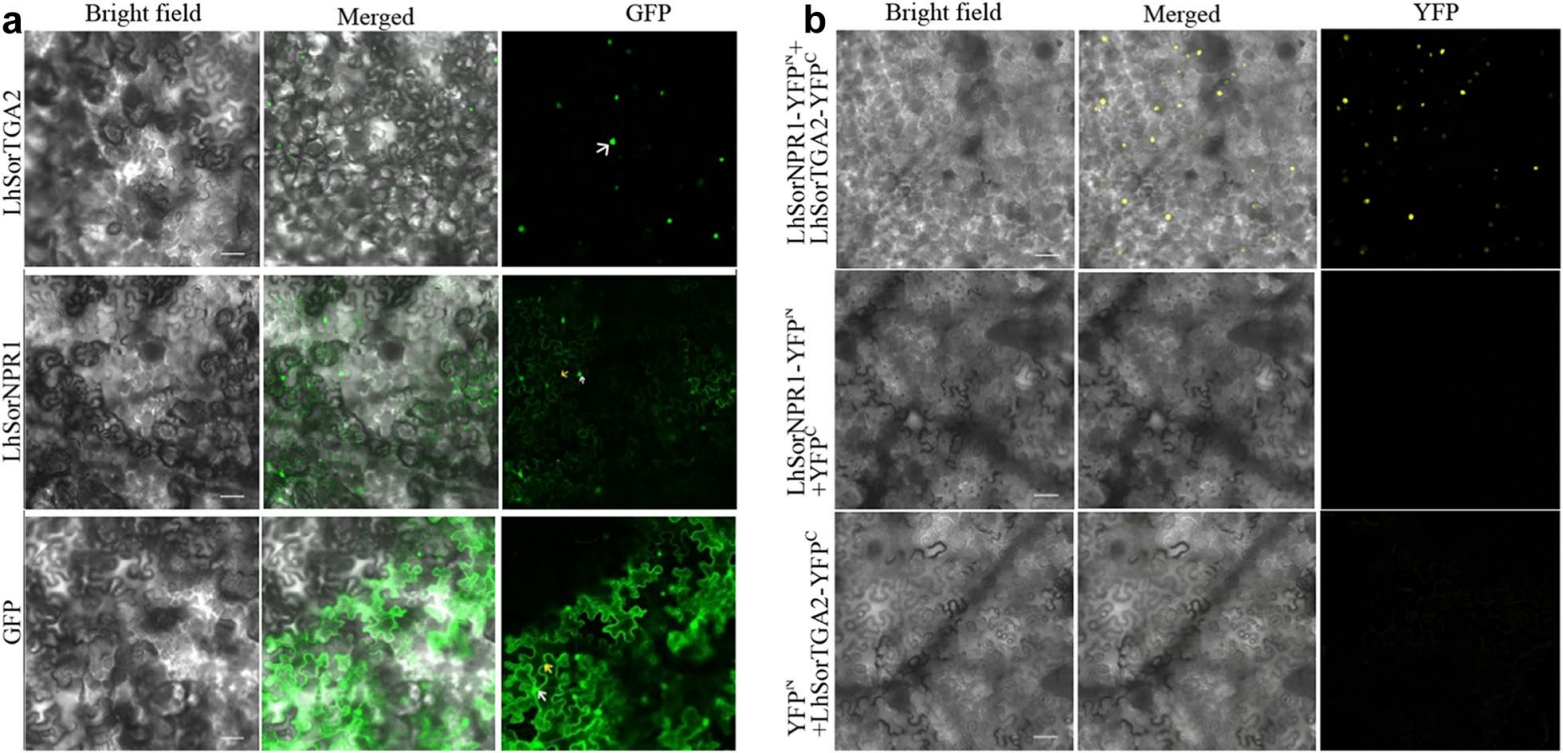
Subcellular localization of LhSorTGA2 and LhSorNPR1, and their interaction validated by BIFC in planta. **a** Localization of LhSorTGA2 and LhSorNPR1 in *N. benthamiana* leaf epidermal cells. **b** BIFC analysis to validate the protein interaction between LhSorTGA2 and LhSorNPR1. LhSorNPR1 was fused to the N-terminal, while LhSorTGA2 was fused to the C-terminal half of YFP protein. Yellow fluorescence indicated protein interaction between LhSorTGA2 and LhSorNPR1 in the nuclei. Fluorescence was observed by confocal microscopy, and bright field and merged images were also shown for each observation. White arrows indicate nuclei of epidermal cells, and yellow arrows indicate the cytomembrane. Bar = 100 µm

LhSorTGA2 and LhSorNPR1 (GenBank Accession: KY073343) were fused to the N-terminus of GFP respectively, and the fusion proteins were transiently expressed in *N. benthamiana* leaf epidermal cells under the control of CaMV 35S promoter to examine their subcellular localization. As shown in Fig. 3a, fluorescence of GFP-tagged LhSorTGA2 was predominantly detected in the nucleus. Different from LhSorTGA2, fluorescence of the LhSorNPR1-GFP infusion protein was observed both in the nucleus and at the cytomembrane. Strong green fluorescence was detected throughout the entire cell for the pCAMBIA1302-GFP control as expected. Our observation of subcellular localization of LhSorTGA2 and LhSorNPR1 was in agreement with their corresponding orthologues in *Gladiolus hybridus* from previous report (Zhong et al. 2015).

Protein–protein interaction between NPR1 and TGA2 was first identified in *Arabidopsis*. To test whether the interaction between LhSorNPR1 and LhSorTGA2 exists or not, we performed BIFC experiment by co-expressing LhSorNPR1-YFP^N^ and LhSorTGA2-YFP^C^ transiently in *N. benthamiana* leaf epidermal cells. As shown in Fig. 3b, strong fluorescence was detected in the LhSorNPR1-YFP^N^ and LhSorTGA2-YFP^C^ co-infiltration treatment, indicating reconstitution of YFP fluorophore by the interaction between LhSorNPR1 and LhSorTGA2. In contrast, co-infiltration of LhSorNPR1-YFP^N^ and YFP^C^ or YFP^N^ and LhSorTGA2-YFP^C^, did not produce any detectable fluorescent signal. These findings indicate that LhSorNPR1 interacts with LhSorTGA2.

### Confirmation of LhSorNPR1–LhSorTGA2 interaction by Y2H

**Fig. 4:**
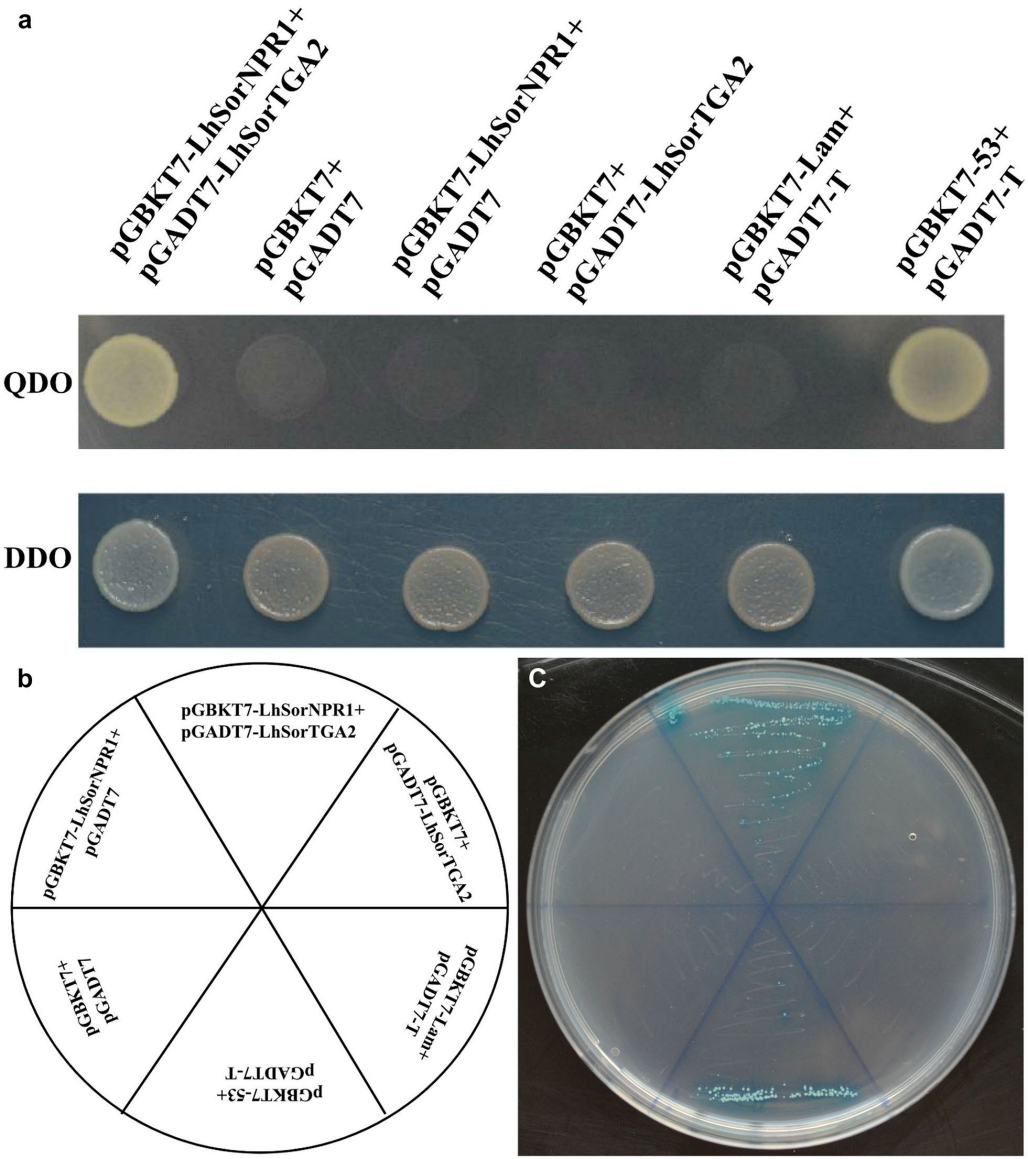
LhSorNPR1 and LhSorTGA2 protein interaction assessed by the yeast two hybrid assay. **a** Yeast AH109 cells co-transformed with bait and prey vectors were grown on double dropout (DDO, SD/-Leu/-Trp) and quadruple dropout (QDO, SD/-Ade/-His/-Leu/-Trp) mediums. **b** The diagram indicates the corresponding co-transformants for the assay in **c**. **c** Transformants were streaked on QDO medium supplemented with 40 µg/ml X-α-Gal. pGBKT7-53 and pGADT7-T co-transformant was used as the positive control. Yeast cells co-transformed with pGBKT7 and pGADT7, pGBKT7-LhSorNPR1 and pGADT7, pGBKT7 and pGADT7-LhSorTGA2, or pGBKT7-Lam and pGADT7-T were used as negative controls

To further confirm the interaction between LhSorTGA2 and LhSorNPR1, the prey plasmid pGADT7-LhSorTGA2 and the bait plasmid pGBKT7-LhSorNPR1 were co-transformed into AH109 competent yeast cells. Transformants were selected on DDO and QDO mediums, and protein–protein interaction between LhSorTGA2 and LhSorNPR1 was identified (Fig. 4a). In contrast, no positive interaction was detected in a series of co-transformants used as negative controls. All co-transformants were streaked on QDO medium supplemented with X-α-Gal chromogenic substrate, and blue colonies were only observed for the pGADT7-LhSorTGA2 and pGBKT7-LhSorNPR1 co-transformant and the positive control (pGADT7-T and pGBKT7-53 co-transformant) (Fig. 4b ,c).

### Expression analysis of *LhSorTGA2* in different tissues and in response to pathogen infection and various hormone treatments

**Fig. 5:**
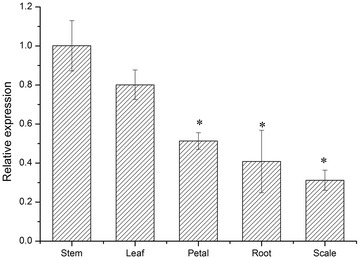
Tissue-specific expression of *LhSorTGA2* in oriental hybrid lily cv. Sorbonne. The expression of *LhSorTGA2* in stem was used as calibrator (designated as 1.0) to determine the relative expression of the target gene in different tissues. Relative expression was calculated using REST 2009 software, and the values were presented as mean ± standard deviation (SD) for three replicates. Bars labeled with an asterisk indicate significant differences from the calibrator at P < 0.05 (the REST statistical randomization test)

Expression of *LhSorTGA2* was detected in all the five tissues explored, but the transcript abundance varied from tissue to tissue (Fig. 5). The transcript levels of *LhSorTGA2* observed in leaf, petal, root, and scale, were all lower than that in stem, making stem the most abundant tissue expressing *LhSorTGA2*.

**Fig. 6:**
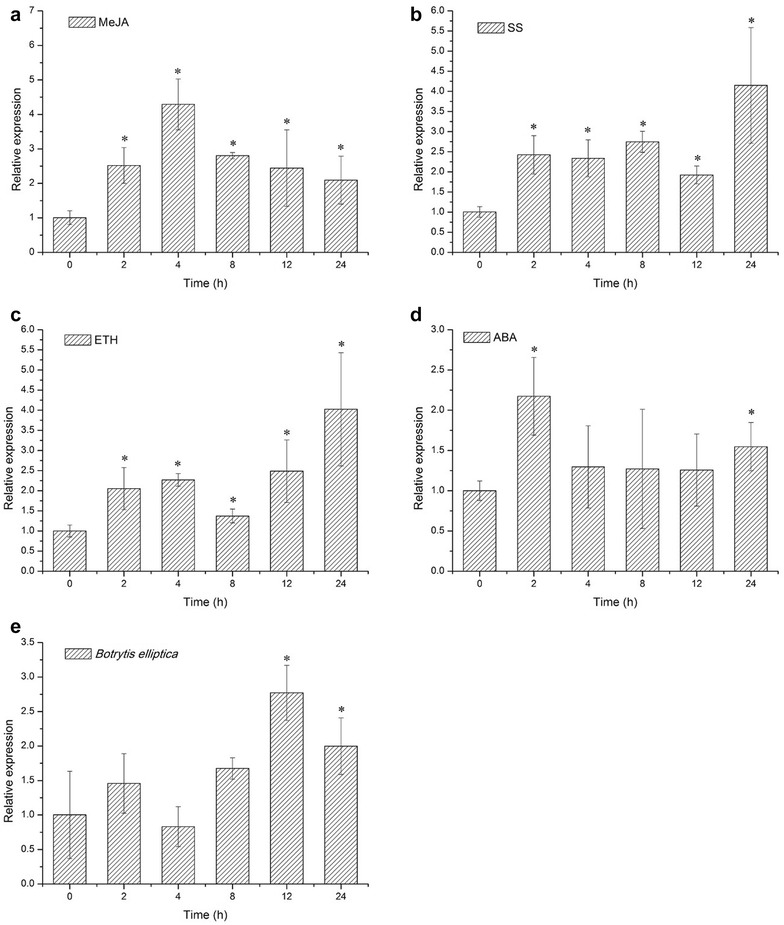
*LhSorTGA2* expression in respond to **a** methyl jasmonate (MeJA), **b** sodium salicylate (SS), **c** ethephon (ETH), **d** abscisic acid (ABA) and **e** *Botrytis elliptica* inoculation treatments. Foliar spray of MeJA, SS, ETH, ABA, or Spores of *B. elliptica* (8 × 10^5^ conidia/ml), was applied to 1-month-old lily seedling. Leaves were sampled 0, 2, 4, 8, 12, 24 h post treatments to explore *LhSorTGA2* expression. Expression at 0 h was used as the calibrator (designated as 1.0) to determine the relative expression of the target gene at different time points. Values are shown as mean ± standard deviations (SD) for three replicates. Bars labeled with an asterisk indicate significant differences from the control (0 h) at P < 0.05 (the REST statistical randomization test)

To investigate *LhSorTGA2* expression in respond to pathogen infection or hormone treatments, transcript levels of *LhSorTGA2* were quantified after subjected to different treatments (MeJA, SA, ETH, ABA, and *B. elliptica* infection). As shown in Fig. 6a, when subjected to MeJA treatment, *LhSorTGA2* transcription was significantly induced, reaching its peak level 4 h after treatment. Foliar spray of SA also stimulated the accumulation of *LhSorTGA2*, resulting in a 4.15 times increase of *LhSorTGA2* transcript level 24 h after treatment (Fig. 6b). Following the ETH treatment, *LhSorTGA2* was up-regulated 4.02 times the control level (Fig. 6c). For the ABA treatment, *LhSorTGA2* expression was rapidly induced 2 h post treatment, reaching a level 2.17 times that of in the control. When exposed to pathogenic fungus *B. elliptica*, the expression of *LhSorTGA2* was also up-regulated, reaching its highest level 12 h after treatment (by a factor of 2.77 compared to the control). Up-regulation of *LhSorTGA2* in respond to fungal infection and hormone treatments suggested that LhSorTGA2 might involve in diverse defense pathways regulated by multiple phytohormones.

## Discussion

Transcriptional reprogramming during SAR is predominantly mediated by NPR1. The interactions between NPR1 and TGA family proteins modulate expression of *PR* genes during SAR. bZIP TFs in *Arabidopsis* are subdivided into 10 groups (A–I and S), among which group D proteins are crucial participants in response to pathogen infection (Jakoby et al. 2002). Previous survey of the *Arabidopsis* genome identified 10 members of the group D bZIP TFs (TGA family proteins), of which seven have been confirmed to exist interaction with NPR1 (Kesarwani et al. 2007).

Lilies are one of the most important floriculture crops in cut flower market and are valued for their elegant flowers emitting fragrance. However, a lack of genome information hinders the genetic improvement targeting genes of interest, for instance, genes related to ornamental traits or disease resistance. Species in the genus *Lilium* have huge genomes (around 36 GB), which makes sequencing and assembly of their genomes a technical challenge (Du et al. 2017). In light of the constraint in availability of the genomic data, RNA-seq seems to be the most practical approach to profile the transcriptional change of genes of interest in lily. In the present study, the partial coding sequence of *LhSorTGA2* was retrieved from our previous transcriptome sequencing data, and the *LhSorTGA2* full-length cDNA was obtained by RACE. Subsequent in silicon analysis confirmed the homology of LhSorTGA2 to TGA-like proteins in other plants, such as AtTGA2 (72.23% similarity) in *Arabidopsis* and GhTGA2 (85.55% similarity) in *Gladiolus* hybrid cultivar. Sequence alignment of LhSorTGA2 with TGA proteins in *Arabidopsis* revealed the structural basis for TGA2-mediated transcription control. The leucine zipper domain determines the dimerization specificity of bZIP TFs (Vinson et al. 2006), forming homo- or heterodimeric coiled-coil structures (zipper). The contiguous basic region, which contains a predicted nuclear localization signal (amino acids 143–159 in LhSorTGA2), contacts the DNA. Whereas the glutamine rich acid domains (QI and QII) have been proven to transactivate *PR1* expression in a NPR1-dependent way during SAR (Fan and Dong 2002).

A phylogenetic tree was built to characterize the evolutionary relationship of TGA-like proteins originated from different species. LhSorTGA2 was grouped in subcladed I, together with AtTGA2, AtTGA5 and AtTGA6. These three closely related TGAs have been proposed as transcriptional repressors of *PR1*, which are required for the basal repression of *PR1*. However, in the state of SAR, their biological functions are both redundant and essential, since only the *tga2 tga5 tga6* triple mutant is blocked from induction of *PR1* during SAR (Zhang et al. 2003).

To further investigate the function of LhSorTGA2 and LhSorNPR1, subcellular localization of GFP-tagged proteins was examined using confocal microscopy. As mentioned above, a NLS was predicted in the middle of LhSorTGA2, which probably making it a nucleus-localized protein. Consistent with the prediction, GFP-tagged LhSorTGA2 was predominately detected in the nucleus. A NLS was also predicted in LhSorNPR1 at the C terminus, but presence of GFP-tagged LhSorNPR1 was detected both in the nucleus and at the cytomembrane. Consecutive expression of GFP-tagged AtNPR1 or TcNPR1 in *Arabidopsis* has proven that NPR1 localized in the cytoplasma if the seedlings were not treated with SA (Mou et al. 2003; Shi et al. 2010). However, under SA treatment (in the state of SAR), both AtNPR1-GFP and TcNPR1-GFP fusion proteins were translocated into the nucleus. Agroinfiltration causes immune response. Hence, SAR might be triggered during subcellular localization of GFP-labeled proteins using tabacco transient expression system. Our observation of LhSorNPR1-GFP in the nucleus was consistent with previous reports (GhNPR1 and VvNPR1), which might be caused by the translocation of LhSorNPR1-GFP into the nucleus in the state of SAR during agroinfiltration (Le Henanff et al. 2009; Zhong et al. 2015).

In *Arabidopsis*, interactions between NPR1 and TGA2 clade proteins modulated the expression of *PR1*. To validate the possible interaction between LhSorTGA2 and LhSorNPR1, vectors were constructed to test interaction in two different systems. As shown in Figs. 3 and 4, both BIFC and Y2H tests confirmed the interaction between LhSorTGA2 and LhSorNPR1. However, whether transcription of *PR* genes, especially *PR1*, is regulated by LhSorTGA2–LhSorNPR1 interaction in lily still needs to be confirmed.

Expression of *LhSorTGA2* was detected in all the five tissues (leaf, stem, root, petal and scale) explored, but the transcription levels varied substantially (Fig. 5). TGA2 participated in SA-regulated SAR induction, and the expression of *TGA2* itself has been shown to be responsive to SA in *Arabidopsis* (Fan and Dong 2002). In our case, *LhSorTGA2* expression was responsive to SA, MeJA, ETH, ABA, and *B. elliptica* inoculation treatments, implying diverse roles it might play in defense pathways regulated by different hormones. Phytohormones are tightly linked to physiological processes in plant (Weyers and Paterson 2001). Induction of *LhSorTGA2* in respond to multiple hormones suggested that LhSorTGA2 might relate to physiological processes contributing to plant defense. Although there is still no direct evidence linking expression of *TGA2*-*like* genes to physiological changes under pathologic conditions, AtTGA10 and AtTGA9, two other members of the TGA family in *Arabidopsis*, has been shown to be involved in reactive oxygen species (ROS)-mediated responses to bacterial PAMP flg22 (Noshi et al. 2016). Accumulation of ROS is associated with hypersensitive response (HR) that restricts the spread of pathogens (Zurbriggen et al. 2010). However, whether TGA family proteins in lily modulate disease resistance through ROS accumulation is unknown. Among these TGA proteins, which one is the key regulator involved in ROS-mediated HR response to pathogen? Answers to these questions are mysteries awaited to be uncovered in future research.

## Conclusions

LhSorTGA2, a novel TGA-like protein, was identified as an interacting partner of LhSorNPR1 in lily. Sequence alignment revealed that LhSorTGA2 was a member of the group D bZIP transcription factors, which featured with a conserved bZIP domain and two glutamine rich acid domains. *LhSorTGA2* was differentially expressed in various tissues, and its expression was responsive to SA, MeJA, ETH, ABA, and *B. elliptica* inoculation treatments. However, whether transcription of *PR* genes is modulated by the interaction between LhSorTGA2 and LhSorNPR1 still needs to be tested.

## Additional files

**Additional file 1: Figure S1.** Cloning of the *LhSorTGA2* gene by RACE. **a** partial coding sequence of *LhSorTGA2*; **b** 5′ *LhSorTGA2* RACE PCR products; **c** 3′ *LhSorTGA2* RACE PCR products; **d** The *LhSorTGA2* open reading frame amplified. M: DNA marker.

**Additional file 2: Table S1.** TGA-like protein sequences retrieved from GenBank used for phylogram construction and sequence alignment.

## Abbreviations

SAR: systemic acquird resistance
NPR1: non-expressor of pathogenesis-related genes 1
bZIP: basic leucine-zipper
RACE: rapid amplification of cDNA ends
BIFC: bimolecular fluorescence complementation
Y2H: yeast two-hybrid
QDO: SD/-Ade/-His/-Leu/-Trp
DDO: SD/-Leu/-Trp
ABA: abscisic acid
SA: salicylic acid
SS: sodium salicylate
MeJA: methyl jasmonate
ETH: ethephon
