# Supplementary figures and images for: Characterization of LhSorTGA2, a novel TGA2-like protein that interacts with LhSorNPR1 in oriental hybrid lily Sorbonne
Source: Bot Stud. 2017 Nov 10;58:46. doi: 10.1186/s40529-017-0201-y (PMC5681460; doi:10.1186/s40529-017-0201-y)

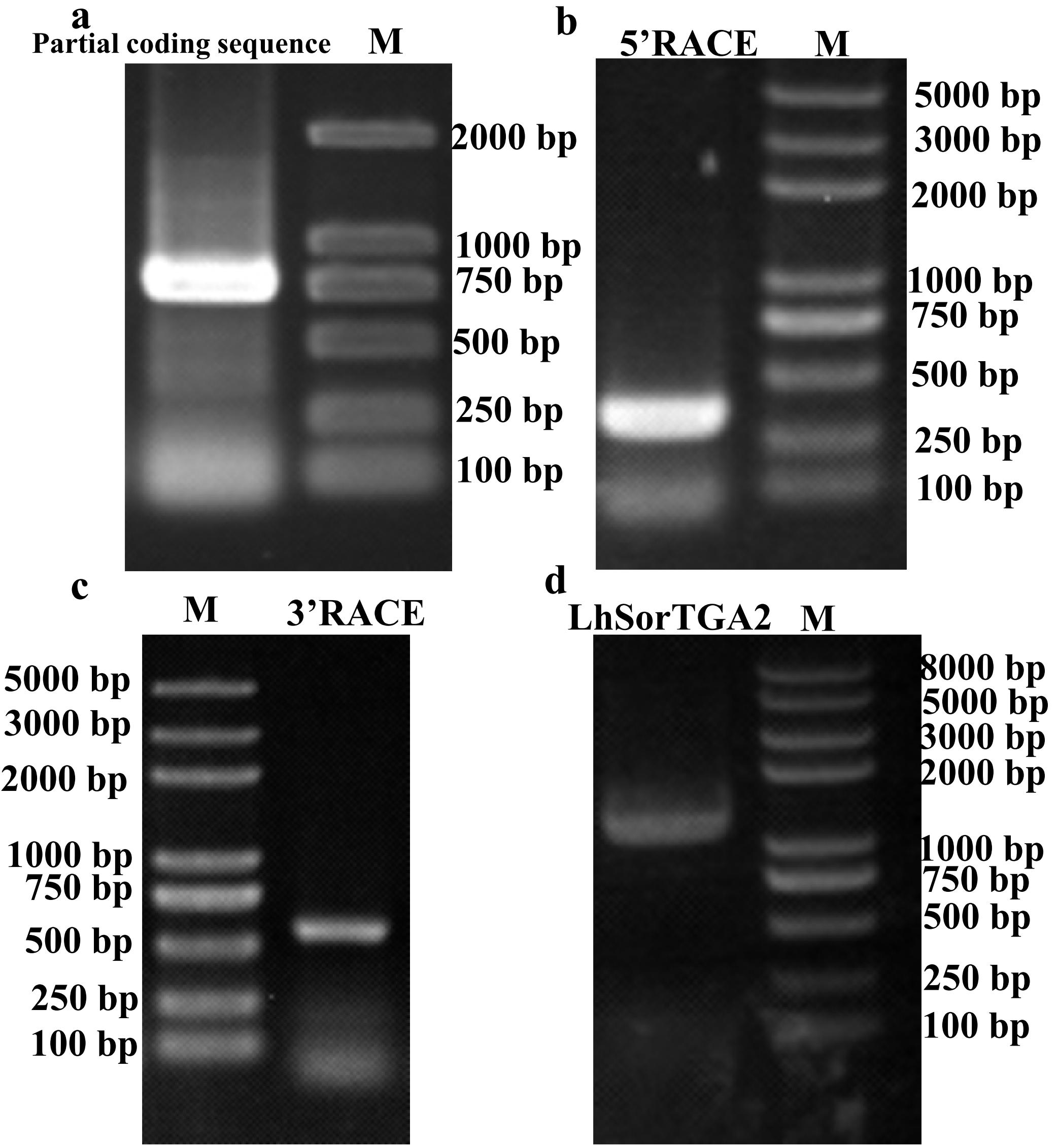

Supplement: Supplementary file 1 — Additional file 1: Figure S1. Cloning of the LhSorTGA2 gene by RACE. a partial coding sequence of LhSorTGA2; b 5′ LhSorTGA2 RACE PCR products; c 3′ LhSorTGA2 RACE PCR products; d The LhSorTGA2 open reading frame amplified. M: DNA marker. [file 40529_2017_201_MOESM1_ESM.jpg]
