# Supplementary material for: Characterization of LhSorTGA2, a novel TGA2-like protein that interacts with LhSorNPR1 in oriental hybrid lily Sorbonne
Source: Bot Stud. 2017 Nov 10;58:46. doi: 10.1186/s40529-017-0201-y (PMC5681460; doi:10.1186/s40529-017-0201-y)
Supplement: Supplementary file 2 — Additional file 2: Table S1. TGA-like protein sequences retrieved from GenBank used for phylogram construction and sequence alignment. [file 40529_2017_201_MOESM2_ESM.docx]

| **Indentifier** | **Accession number** | **Species** |
| --- | --- | --- |
| AtTGA1 | NP_851273 | *Arabidopsis thaliana* |
| AtTGA2 | NP_196312 | *A. thaliana* |
| AtTGA3 | NP_564156 | *A. thaliana* |
| AtTGA4 | NP_196565 | *A. thaliana* |
| AtTGA5 | NP_196313 | *A. thaliana* |
| AtTGA6 | NP_974292 | *A. thaliana* |
| AtTGA7 | NP_565162 | *A. thaliana* |
| AtPAN | NP_177031 | *A. thaliana* |
| AtTGA 9 | NP_001321503 | *A. thaliana* |
| AtTGA10 | NP_001190244 | *A. thaliana* |
| GhTGA2 | AGI17587 | *Gladiolus hybrid cultivar* |
| DcTGA2.3 | XP_020698027 | *Dendrobium catenatum* |
| PeTGA2.3 | XP_020583361 | *Phalaenopsis equestris* |
| AtrTGA2.3 | XP_006832856 | *Amborella trichopoda* |
|  |  |  |
|  |  |  |
|  |  |  |
|  |  |  |
|  |  |  |
|  |  |  |
|  |  |  |
|  |  |  |
|  |  |  |
|  |  |  |
|  |  |  |
|  |  |  |
|  |  |  |
|  |  |  |
|  |  |  |
|  |  |  |
|  |  |  |

**Table S1** TGA-like protein sequences retrieved from GenBank used for phylogram construction and sequence alignment.
